# Influence of repeated implant‐abutment manipulation on the prevalence of peri‐implant diseases in complete arch restorations. A retrospective analysis

**DOI:** 10.1111/cid.13381

**Published:** 2024-09-11

**Authors:** M. Krebs, L. Greilich, P. Weigl, P. Hess, I. Dahmer, A. Begić

**Affiliations:** ^1^ Department of Postgraduate Education Goethe University, Carolinum Frankfurt Germany; ^2^ Privat Practice Dr. Krebs & Colleagues Alzey Germany; ^3^ Department of Oral Surgery and Implantology Goethe University, Carolinum Frankfurt Germany; ^4^ Institute of Biostatistics and Mathematical Modeling Goethe University, Carolinum Frankfurt Germany; ^5^ Center of Dentistry and Oral Medicine Goethe University Frankfurt Germany

**Keywords:** abutment replacements, immediately loaded, one‐abutment one‐time, peri‐implantitis, prevalence

## Abstract

**Purpose:**

To evaluate the effects of repeated abutment manipulation on the prevalence of peri‐implant diseases.

**Materials and Methods:**

A total of 27 edentulous patients (*n* = 108 implants) immediately restored with double‐crown retained implant‐supported prostheses were identified for this retrospective study. The test included the one‐abutment, one‐time care concept (*n* = 18 patients, *n* = 72 implants, OAOT) and the control abutment replacement (*n* = 9 patients, *n* = 36 implants, AR). A mixed effects model regression was conducted for the variable diagnosis (healthy, peri‐implant mucositis, and peri‐implantitis) with predictors abutment replacement (presence/absence), number of abutment replacement, category of keratinized mucosa (KM) (2 < KM ≥2 mm), and radiographic bone loss (BL).

**Results:**

After 3–15 years (mean 10.2 ± 2.8 years), the prevalence of peri‐implant mucositis and peri‐implantitis in patients in the AR group was 11.1% and 88.9%, corresponding to 22.2% and 55.6% at the implant level, respectively. In OAOT group, none of the implants showed peri‐implant mucositis, whereas the prevalence for peri‐implantitis at patient and implant level amounted to 5.6% and 5.6%, respectively. The increased number of abutment replacements was significantly associated with the increased probability to diagnose peri‐implant mucositis and peri‐implantitis (OR: 6.13; 95% CI [2.61, 14.39]) (*p <* 0.001), whereas the presence of keratinized mucosa was not founded as a significant cofounder. The estimated mean BL in AR group was 1.38 mm larger than in OAOT group (*p =* 0.0190).

**Conclusions:**

The OAOT concept was associated with a lower prevalence of peri‐implant diseases.

**Summary Box**

**What is known**

- Several studies and systematic reviews describe significant bone loss after repeated abutment manipulations.

**What this study adds**

- This study is the first study reporting on the prevalence of peri implant diseases as mucositis and peri‐implantitis in cases with and without repeated abutment manipulation.

## 1 INTRODUCTION

Peri‐implant diseases are biofilm‐related inflammatory pathological conditions affecting the tissues surrounding dental implants in function.^1^ A crucial element of current case definitions to discern peri‐implant mucositis and peri‐implantitis relates to the presence or absence of a radiographic bone loss following the initial remodeling of the crestal bone. In the presence of peri‐implant mucosal inflammation, this progressive bone loss is ideally verified based on previous examination data (i.e., baseline radiographs), but may also be identified by using threshold values for bone levels (i.e., >3 mm).^2^

While the initial bone remodeling following implant placement is considered a physiological event,^3^ its extension and subsequently the exposure of endosseous components may predispose implant sites to the occurrence of peri‐implant diseases.^1^ Numerous factors, such as the thickness of the peri‐implant mucosa,^4^ prosthetic connections,^5^ as well as the positioning of the implant, have been associated with the extent of initial crestal bone level changes.^6^, ^7^ While the overall effect of the macro/microdesign and material of the abutment was shown to be minimal,^8^ there is evidence describing that the frequency of the abutment manipulation may have a major influence on marginal bone levels.^9^, ^10^ In particular, at 6 months, an abutment change was associated with a significantly higher radiographic bone loss when compared with immediately delivered final abutments (−1.24 ± 0.79 vs. −0.61 ± 0.40 mm).^10^ Histologically, a repeated abutment exchange was associated with a more apical extension of the junctional epithelium, thus resulting in a displacement of the subepithelial connective tissue zone.^9^ In a systematic review, the “one‐abutment one‐time principle” has also been associated with a tendency towards a lower occurrence of biological complications.^11^ However, the included studies inconsistently reported on the prevalence of peri‐implant diseases and did not consider established case definitions.

It was hypothesized, that multiple abutment replacements are associated with a higher prevalence of peri‐implant diseases.

Therefore, the present retrospective analysis further aimed at evaluating the effects of abutment manipulation (i.e., one‐abutment one‐time vs. conventional multiple abutment replacements) on the prevalence of peri‐implant diseases.

## 2 MATERIALS AND METHODS

### 2.1 Study design and participants

This retrospective analysis included a total of *n* = 27 patients with edentulous mandibles (female: 16; male: 11; mean age: 74.2 ± 9.3 years) rehabilitated with 4 intraforaminal implants (in total *n* = 108 implants). All patients were in need of an implant‐supported restoration in the mandible and received established and standardized treatment protocols at the Department of Oral Surgery and Implantology, Goethe University, Frankfurt, Germany, between 1998 and 2015.

The case definition for peri‐implantitis included BOP and progressive radiographic bone loss (i.e., >1.50 mm as double of the measurement error of calibrated panoramic radiographs).^12^ A newer case definition was published in 2018 after ethical approval.^2^ Peri‐implant mucositis was defined by the presence of BOP and absence of a radiographic bone loss. No inflammation signs were detected in health.

The study protocol was approved (No. 19‐242) by the ethics committee of the Goethe University, Frankfurt, Germany based on the Helsinki Declaration of 1975 (revised in August 2018). The present reporting considered the checklist items as proposed in the STROBE statement.^13^

### 2.2 Inclusion criteria

The patients were identified by 2 investigators (G.L., M.K.) in a database (ImpDat, Kea‐Software‐GmbH, Pöcking, Germany) and selected when all the following inclusion criteria were met: (a) edentulous mandible, (b) four interforaminally, subcrestally placed implants with the same type of a morse taper connection (Ankylos®, Dentsply Sirona GmbH, Bensheim, Germany), (c) same surgical technique (i.e., elevation of mucoperiosteal flaps; no application of bone grafts), (d) baseline (XR0) and follow‐up panoramic radiographs (XR1) after at least 3 years available, (e) immediate restoration with a removable denture with double‐crown technique, (f) attendance of yearly routine implant maintenance appointment at the Department of Oral Surgery and Implantology, (g) recording of BOP scores, and (h) availability of all data.

### 2.3 Treatment procedures

**FIGURE 1:**
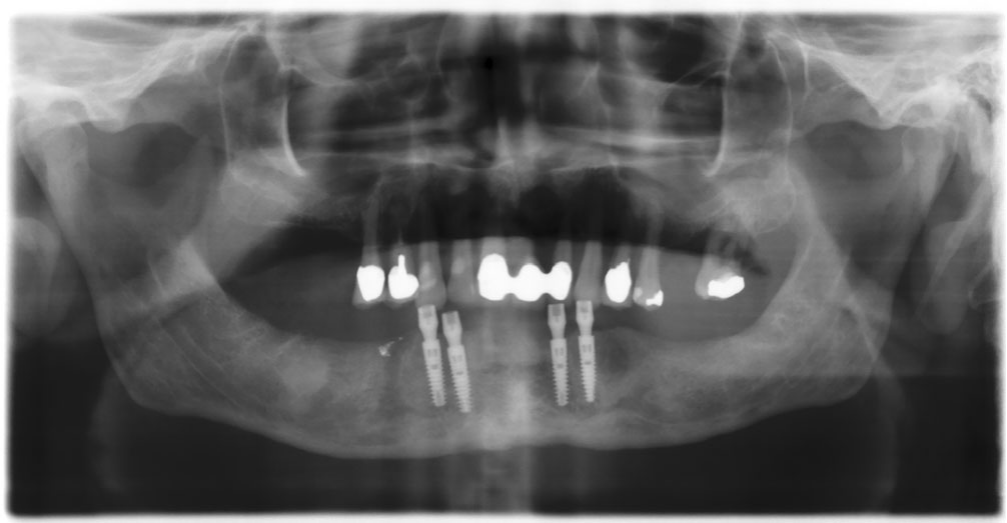
One‐abutment one‐time group picture after implant placement.

**FIGURE 2:**
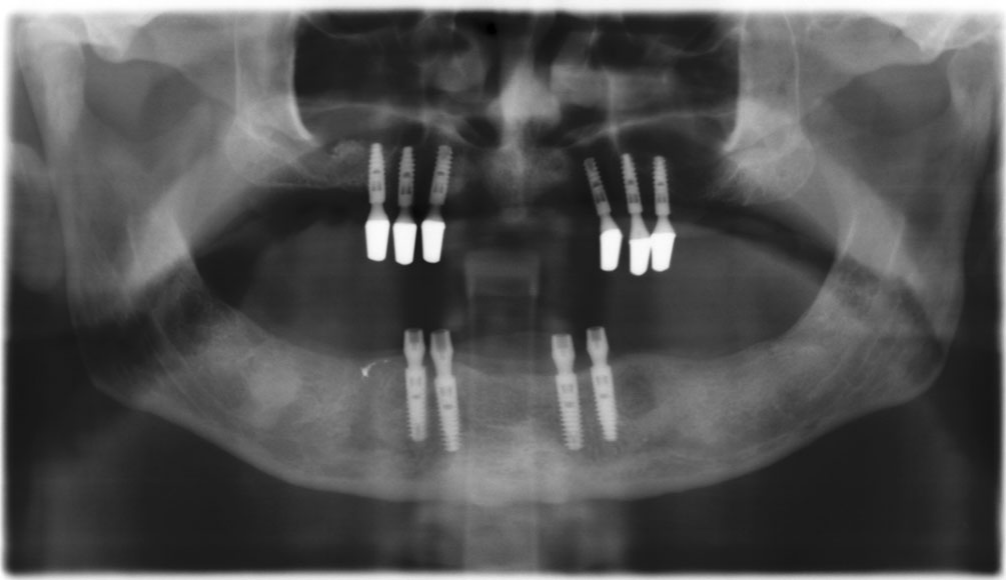
One‐abutment one‐time group last X‐ray control after 11 years.

**FIGURE 3:**
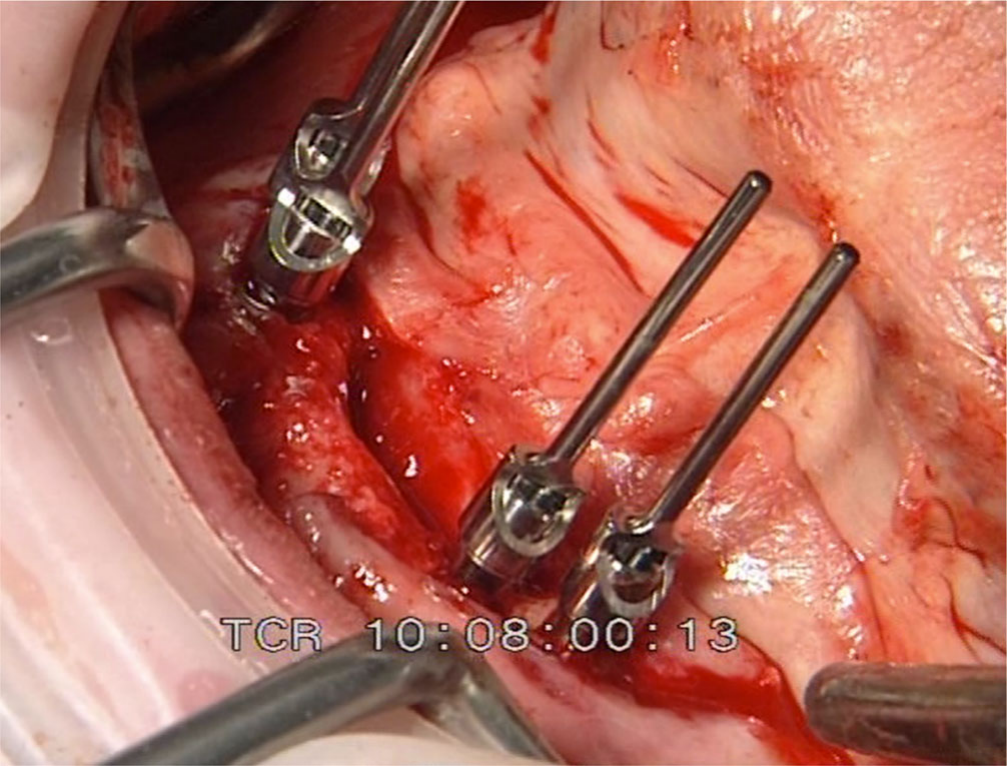
One‐abutment one‐time Group and AR group implant placement, abutment placement and parallelization.

**FIGURE 4:**
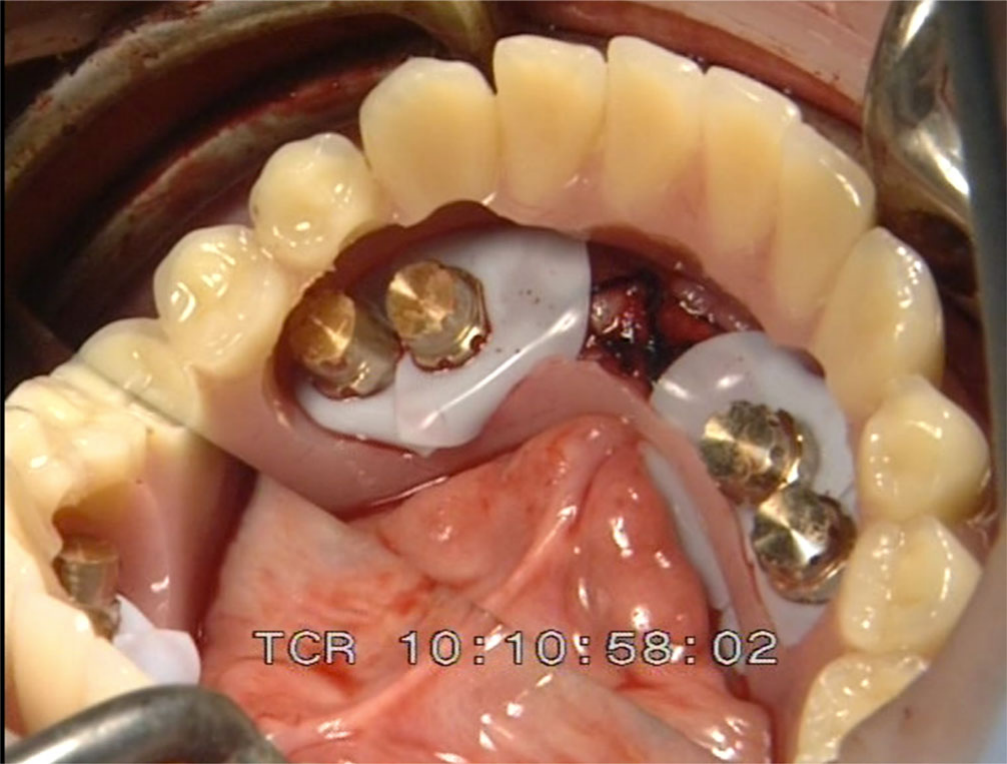
One‐abutment one‐time group and AR group prefabricated secondary crowns glued into temporary denture.

**FIGURE 5:**
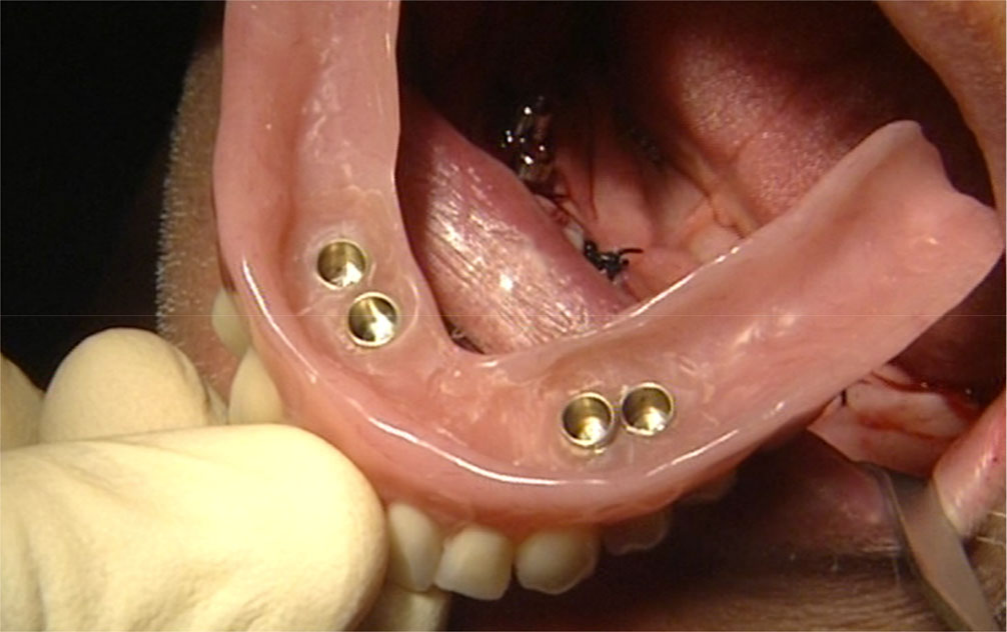
One‐abutment one‐time group and AR group finalized temporary denture for immediate loading.

**FIGURE 6:**
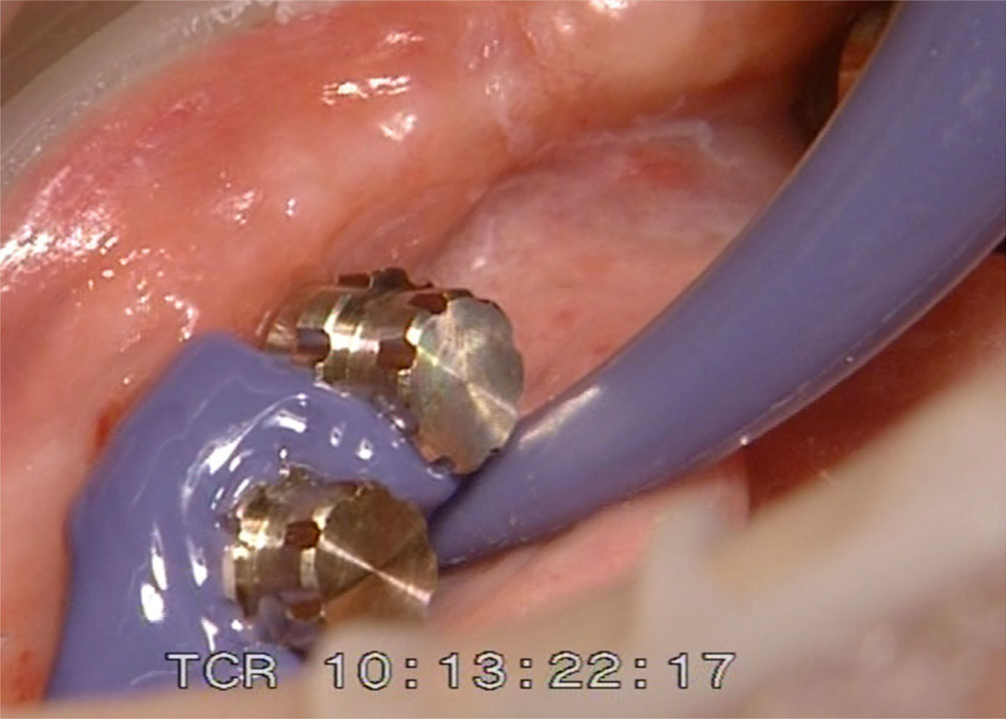
One‐abutment one‐time group pick‐up impression of new prefabricated secondary crowns for the final denture without abutment exchange.

**FIGURE 7:**
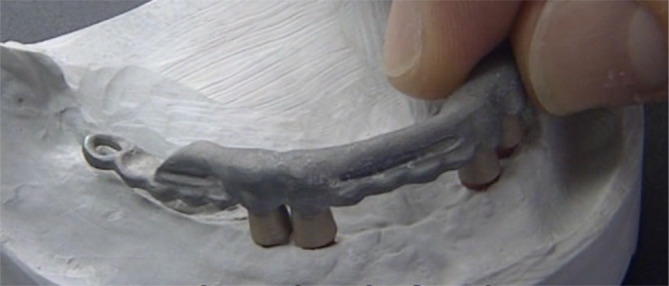
One‐abutment one‐time group final frame work.

**FIGURE 8:**
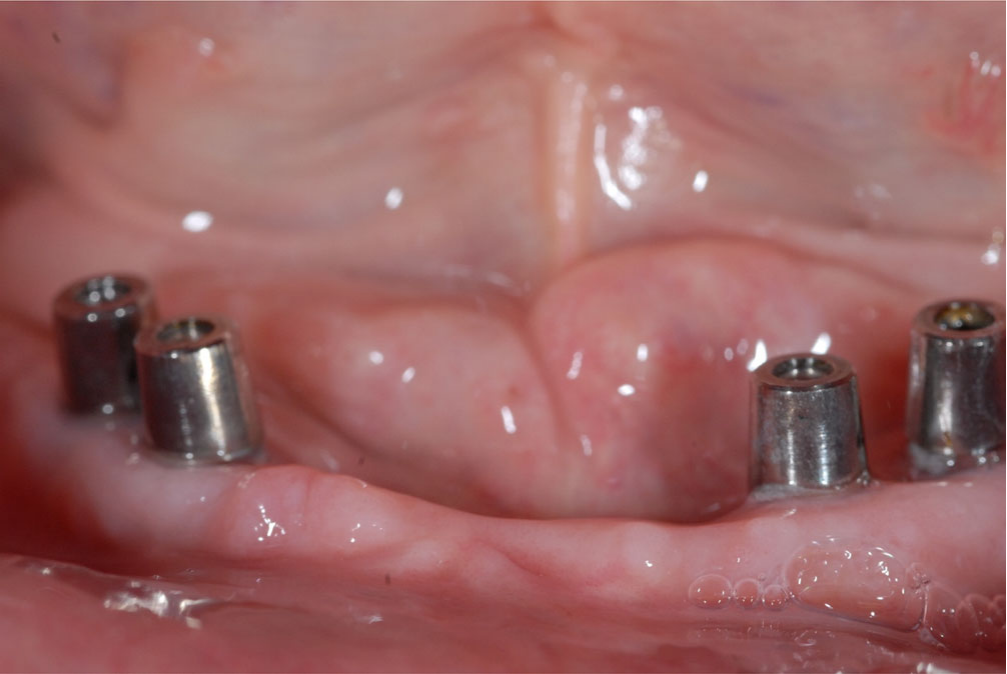
One‐abutment one‐time intraoral view of the Syncone abutments.

In the test group, the one‐abutment, one‐time care concept (OAOT) was applied. For this purpose, patients were selected who were prosthetically restored according to the SynCone (Dentsply Sirona GmbH, Bensheim, Germany) immediate restoration concept.^14^ Four interforaminally placed dental implants in the edentulous mandible are immediately loaded with definitive prefabricated conical abutments serving as a primary crown for a denture's telescopic retention mode. Prefabricated secondary caps are glued into a complete resin denture serving as an implant‐borne removable long‐term provisional bridge. After osseointegration and due to a risk of denture fractures (as no framework is initially integrated), usually within 1 year, a new denture was manufactured with a cobalt–chrome framework on a base of an abutment level impression and new secondary caps without any abutment replacements (Figures 1, 2, 3, 4, 5, 6, 7, 8).

**FIGURE 9:**
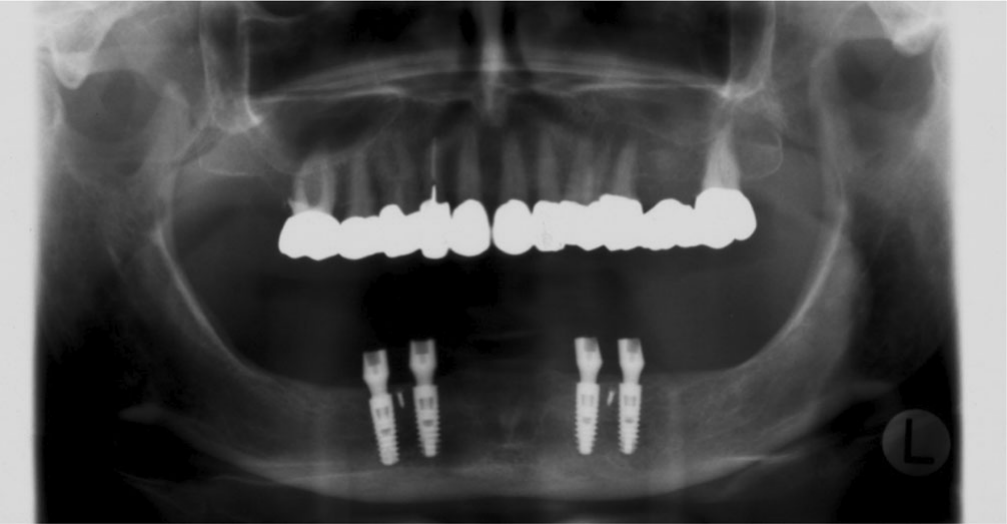
AR group  picture after implant placement (same protocol as OAOT group).

**FIGURE 10:**
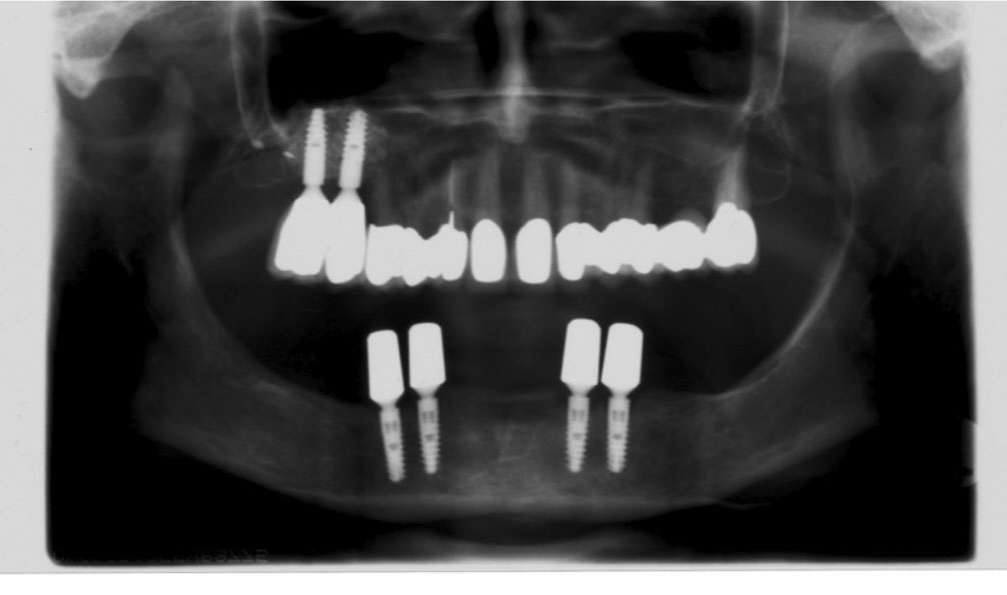
AR group  X‐ray after seating of the final double crown abutments.

**FIGURE 11:**
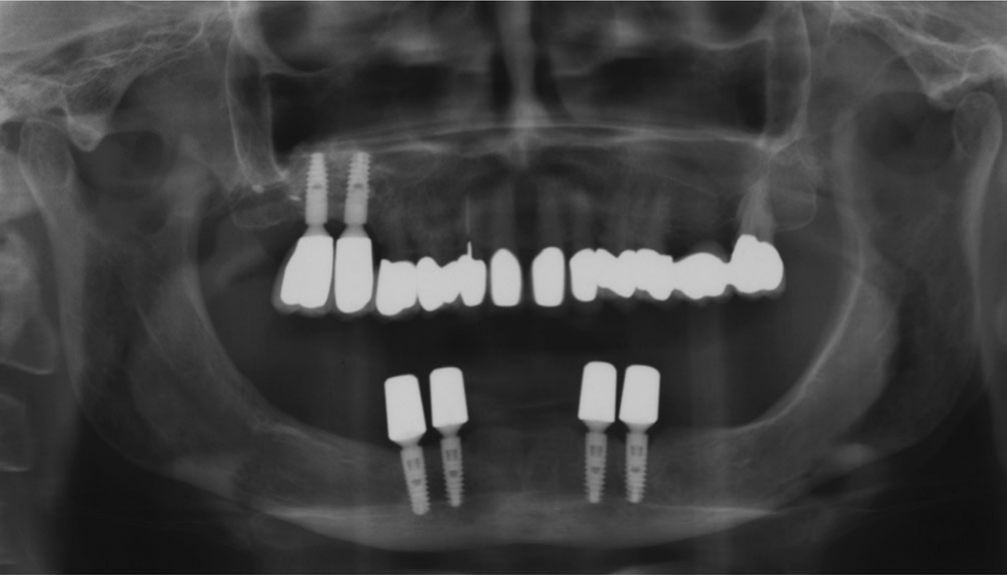
AR group X‐ray 13 years after loading.

**FIGURE 12:**
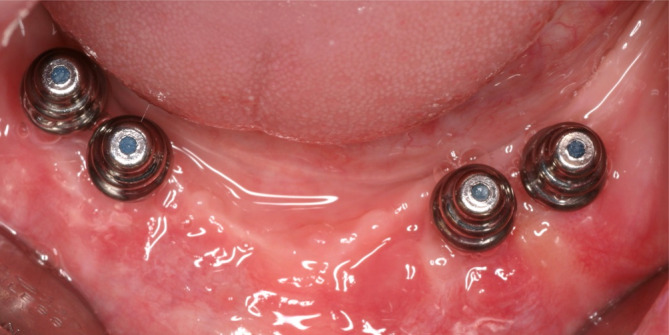
AR group impression posts inserted for the final impression after healing (abutment change number one).

**FIGURE 13:**
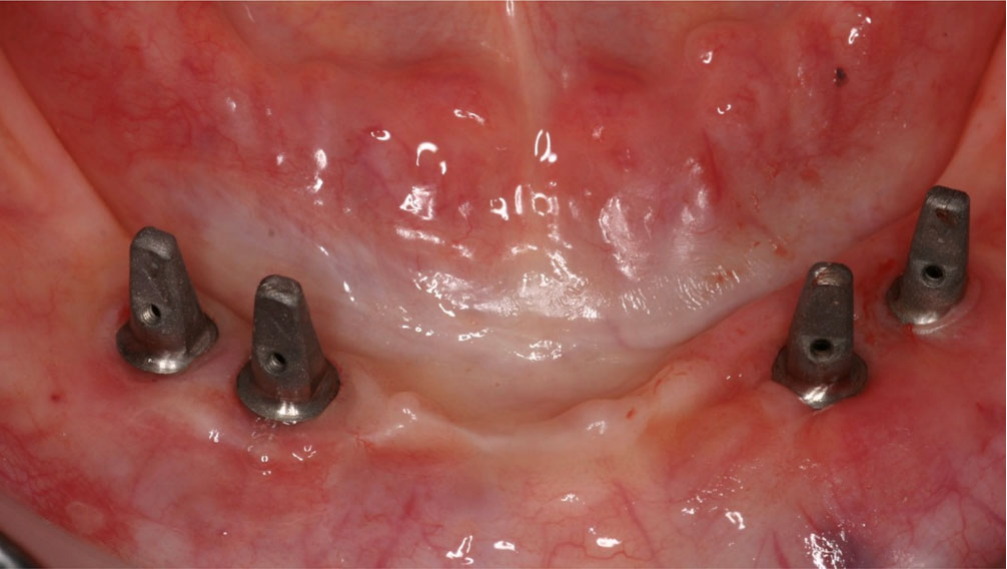
AR group final abutments inserted.

**FIGURE 14:**
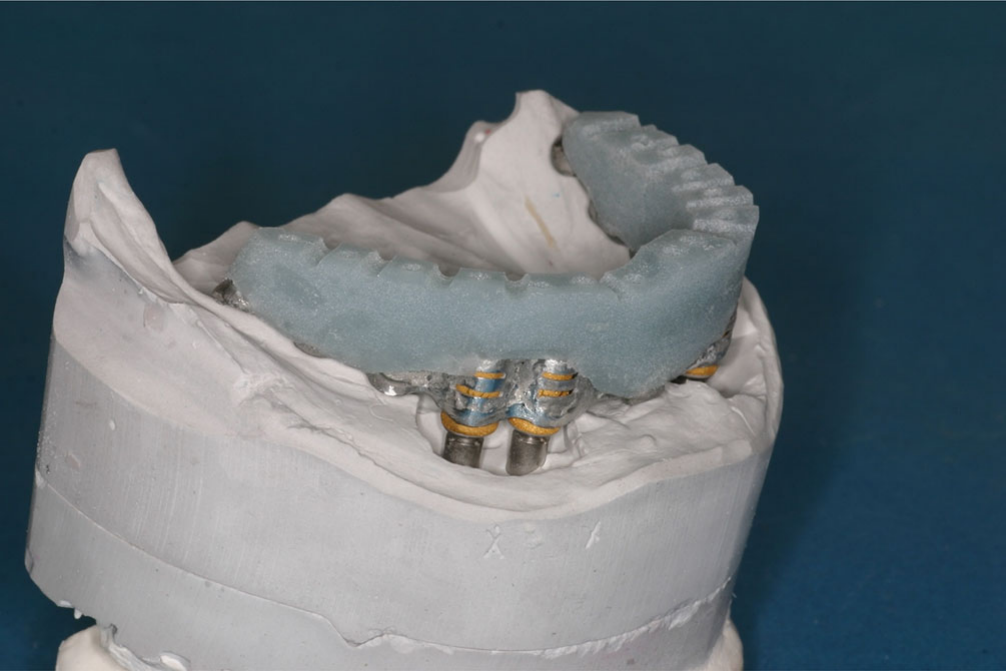
AR group final framework, primary and secondary crown on master cast.

**FIGURE 15:**
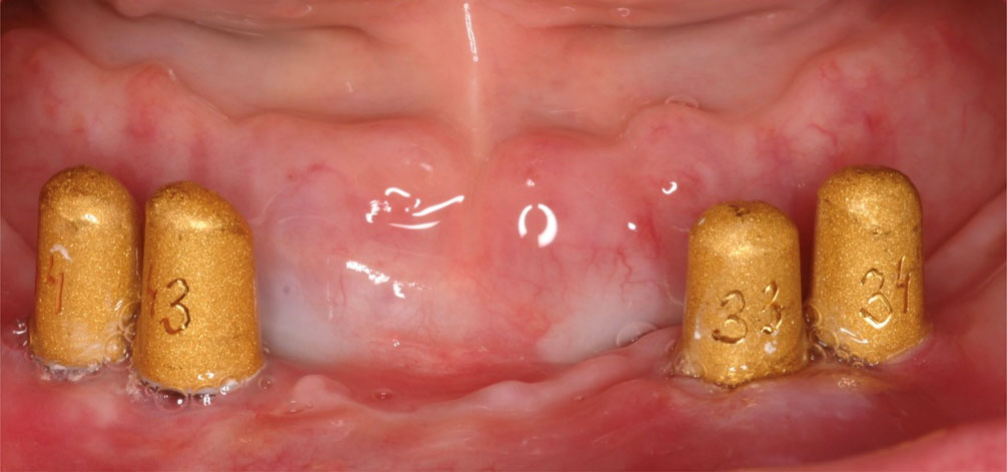
AR group final primary and secondary crown cemented.

**FIGURE 16:**
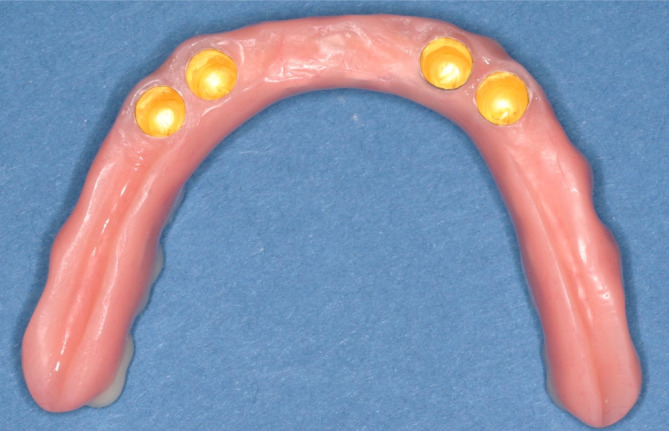
AR finalized Galvano denture.

In the control group (abutment replacement group [AR]), patient treatment starts identical to the test group with SynCone abutments installed on the day of surgery for immediate loading. In these patients, the reconstruction was changed from the prefabricated SynCone parts to lab‐made zirconia primary crowns with electro‐formed gold secondary crowns. Due to this, an impression on implant level and several abutment replacements (bite relation, try‐in) were necessary^15^ (Figures 9, 10, 11, 12, 13, 14, 15, 16).

### 2.4 Assessment of clinical and radiographical parameters

The following patient‐related data were analyzed: (1) age, (2) gender. Implant related (1) midvestibular keratinized mucosa, (2) bleeding on probing (BOP), evaluated as present if the bleeding was evident within 30 s after probing, (3) implant location, (4) implant length, (5) implant insertion depth, (6) loading time, measured from implant insertion and immediate prosthetic restoration to last follow‐up visit, (7) frequency of abutment replacement, (8) interproximal bone level measured at X‐ray image from the day of implant placement, and immediate prosthetic restoration (XR0: radiographic baseline) and follow‐up X‐ray (XR1).

### 2.5 Radiographic assessment

**FIGURE 17:**
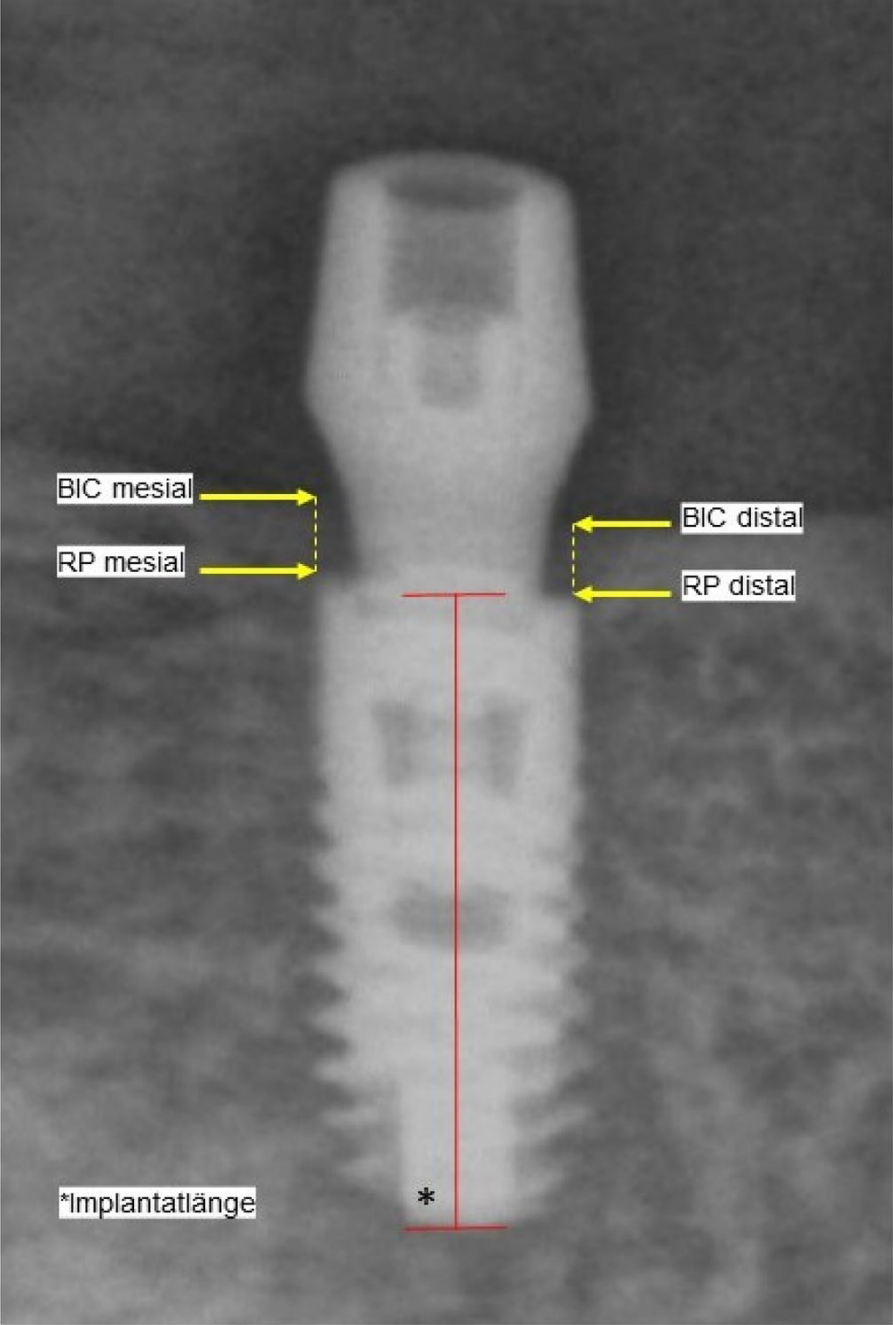
Measurement of interproximal bone level (one‐abutment one‐time: mesial 1.45 mm; distal: 1.33 mm; Implant length 11 mm).

All analog panoramic radiographs (Orthophos‐XG3, Dentsply Sirona GmbH, Bensheim, Germany) were digitized by an X‐ray image scanner (Perfection‐V800 Photo; EPSON Deutschland GmbH, Meerbusch, Germany) and digitally measured in the SIDEXIS XG Database (Dentsply Sirona GmbH, Bensheim, Germany) by 2 investigators (G.L., M.K.). Interproximal bone levels were determined according to a previously established procedure.^14^ In brief, the most coronal and the most apical point of the peri‐implant bone crest at mesial and distal sites were marked. The measurement was carried out parallel to the implant axis and were traced perpendicularly at mesial and distal sites. Each length measurement was recalibrated using the known implant lengths. Implant shoulder was defined as the reference zero point. The bone level exceeding the implant shoulder was determined with positive values. The bone level apical of the implant shoulder received negative values (Figure 17). All measurements were carried out twice by the same operator to exclude variances (L.G.), and the examiner was calibrated for the measurements. The evaluation with the Student's *t*‐test showed no significant measurement differences at the 5% level. An averaged value was used for further calculation so that each implant received a mesial and a distal measured value.

To determine the long‐term radiographic bone loss, a difference in bone levels between XR0 and XR1 was compared (= XR0 – XR1). A positive value was defined as bone loss, a negative value as bone gain. Finally, only the higher bone difference of the mesial or distal implant side was tracked on one occasion.

### 2.6 Statistical analysis

The statistical analysis was conducted using the software program R (R Core Team, 2021). For each variable and group, descriptive statistics were conducted.

For the statistical analysis, the implant was considered a statistical unit. In order to account for the fact that each patient contributed with four implants, mixed models with the patient as random effects were considered. Univariate and multiple ordinal regressions with mixed models were conducted for the dependent variable diagnosis (healthy/peri‐implant mucositis/peri‐implantitis) with abutment change (presence/absence), number of abutment changes, and category KM (KM <2 mm/KM ≥2 mm) (regarded as a confounder) as predictors. A patient was categorized as diseased if at least one of the four implants was affected by peri‐implantitis. The influence of the group on the radiographical bone loss was investigated by means of a linear regression with mixed models having the group as the fixed effect. Results were rated significant at *p* < 0.05%.

## 3 RESULTS

**FIGURE 18:**
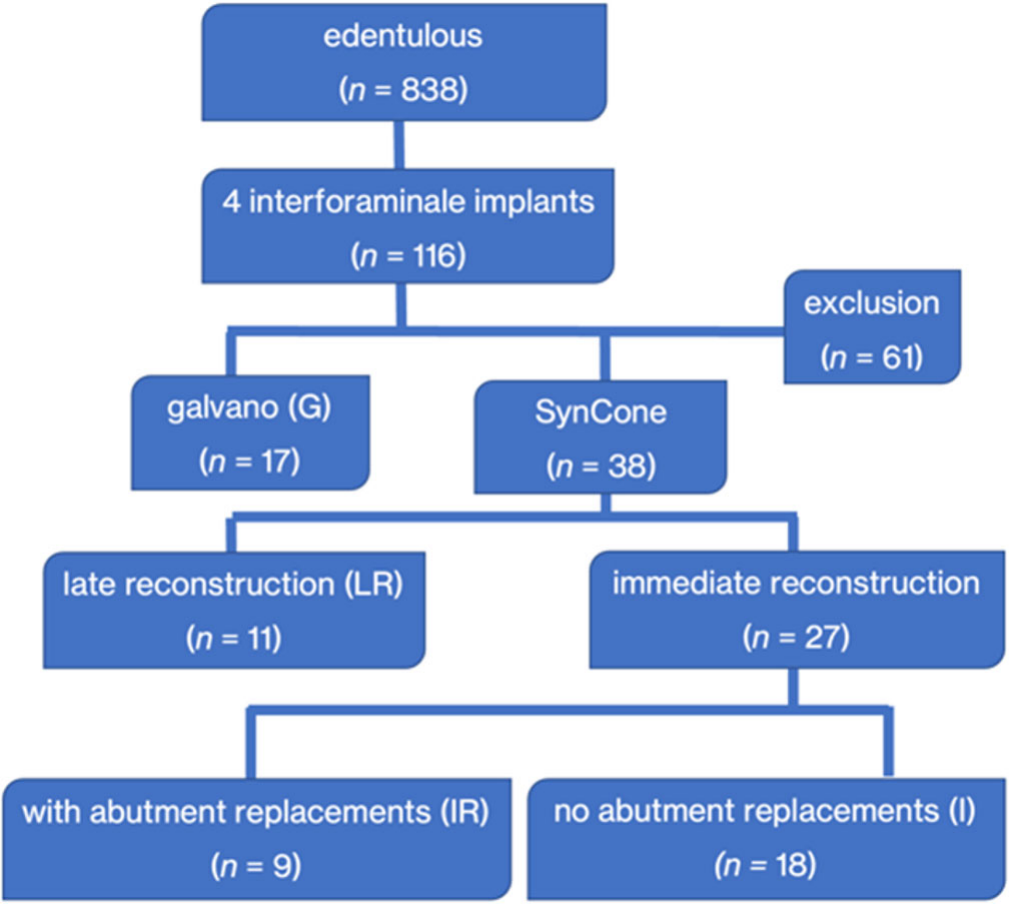
Distribution of the included subjects according to the eligibility criteria.

The records of total *n* = 116 potentially relevant patients with 4 interforaminal implants in the lower jaw were screened. Based on the previously defined inclusion criteria, a total of *n* = 27 patients (female: 16; male: 11; mean age: 74.2 ± 9.3 years) could be considered for the present analysis (Figure 18).

The patient‐related status at baseline, BOP, and the width of keratinized mucosa at last follow‐up, as well as the implant‐related variables and the loading time in different groups, are summarized in Table 1.

**TABLE 1:** Patient and implant characteristics in different groups (*n* = 27 patients, *n* = 108 implants).

Group	OAOT	AR
Gender (*n*, male/female)	8/10	3/6
Age at implant insertion (years, mean ± SD, 95% CI)	62.97 ± 9.53 (58.23, 67.71)	64.99 ± 9.39 (57.77, 72.21)
Implants^a^ (*n*)	72	36
Implant length (*n*, [%, 95% CI])		
9.5 mm	3, (4.2, [0.86, 11.70])	0, (0.0, [0.00, 9.74])
11 mm	40, (55.6, [43.36, 67.28])	24, (66.7, [49.03, 81.44])
14 mm	29, (40.3, [28.88, 52.50])	12, (33.3, [18.56, 50.97])
Depth of implant insertion^b^ (mm, mean ± SD, 95% CI)	1.55 ± 0.90 (1.34, 1.76)	1.66 ± 1.15 (1.28, 2.06)
Keratinized mucosa^c^ (mm, mean ± SD, 95% CI)	3.19 ± 1.73 (2.76, 3.63)	2.02 ± 1.23 (1.57, 2.46)
Category keratinized mucosa (*n*, [%, 95% CI])		
<2 mm	9, (14.1, [6.63, 25.02])	8, (25.0, [11.46, 43.40])
≥2 mm	55, (85.9, [74.98, 93.36])	24, (75.0, [56.60, 88.53])
BOP^c^, ^d^ (*n*, [%, 95% CI])	4, (5.6, [15.34, 13.62])	24, (66, [49.03, 81.44])
Loading time (years, mean ± SD, 95% CI)	9.23 ± 3.23 (7.62, 10.83)	11.22 ± 2.39 (9.38, 13.05)

^a^ Lower jaw, region canine, and first premolar.

^b^ Positive value related to subcrestal insertion.

^c^ Values at last follow‐up.

^d^ Values at implant level. BOP.

In total, 108 subcrestally inserted implants in 27 patients were analyzed. The most common implant length was 11 mm, with 55.6% in OAOT and 66.7% in AR group, respectively. The majority of the implants showed a keratinized mucosa width ≥2 mm, in particular, 85.9% in the OAOT group and 75.0% in the AR groups. BOP was presented at 5.6% of the sites if OAOT protocol was performed, otherwise at 77.8% of the sites. None of the included implants presented suppuration.

### 3.1 Prevalence of peri‐implant diseases and the frequency of abutment replacement

The prevalence of peri‐implant tissue health and disease by groups is presented in Table 2. Based on the given case definitions, the peri‐implant conditions were healthy in 94.4% and 0% for the OAOT and AR groups, respectively. A total of 5.6% and 88.9% of the patients were diagnosed with peri‐implantitis in the aforementioned groups. Peri‐implant mucositis was diagnosed only in the AR group with 11.1%. At the implant level, the corresponding values amounted to 94.4% and 22.2% (healthy), 0% and 22.2% (peri‐implant mucositis), and 5.6% and 55.6% (peri‐implantitis) in the OAOT and AR groups.

**TABLE 2:** Prevalence of healthy and peri‐implant diseases at patient and implant level.

	OAOT		AR	
Groups	Patient level *n*, (%, [95% CI])	Implant level *n*, (%, [95% CI])	Patient level *n*, (%, [95% CI])	Implant level *n*, (%, [95% CI])
Healthy	17, (94.4, [72.71, 99.86])	68, (94.4, [86.38, 98.47])	0, (0.0, [0.00, 33.63])	8, (22.2, [10.12, 39.15])
Peri‐implant mucositis	0, (0.0, [0.00, 18.53])	0, (0,0, [0.00, 4.99])	1, (11.1, [0.03, 48.25])	8, (22.2, [10.12, 39.15])
Peri‐implantitis	1, (5.6, [0.01, 27.29])	4, (5.6, [1.53, 13.62])	8, (88.9, [51.75, 99.72])	20, (55.6, [38.10, 72.06])

In the AR group, the mean abutment replacement was 3.28 ± 0.97 (min: 2, max: 6, median: 3).

### 3.2 Predictors related with peri‐implant diseases

The results of a regression analysis are depicted in Table 3. abutment replacement was a significant predictor of the prevalence of peri‐implant diseases (*p <* 0.001). The change in log odds of the diagnosis being in a higher as opposed to a lower category (0 healthy, 1 peri‐implant mucositis, 2 peri‐implantitis) due to the presence of abutment replacement was equal to 9.179. The presence of abutment replacement led to an increased probability of the diagnosis being in a higher category.

**TABLE 3:** Results from the regression analysis (Predictors related with peri‐implant diseases).

Predictor	Odds ratio (OR)	95% CI	*p* value
(AR/OAOT)^a^	9.179	4.6; 2.03 × 10^6^	<0.001
Number of abutment replacement^b^	6.13	2.61; 14.39	<0.001

^a^ Univariate ordinal regression with mixed effects.

^b^ Multiple ordinal regression with mixed effects (with category KM as confounder).

The number of abutment replacements significantly predicted the prevalence of peri‐implant diseases (*p <* 0.001). The change in log odds of the diagnosis being in a higher as opposed to a lower category (0 healthy, 1 peri‐implant mucositis, 2 peri‐implantitis) unit increase of the number of abutment changes was 1.81335. The rising number of abutment replacements led to an increased probability of the diagnosis being in a higher category (OR: 6.13; 95%CI [2.61, 14.39]).

The statistical analysis failed to reveal a significance for the factor keratinized mucosa ([mm ± SD] OAOT: 3.19 ± 1.73; AR: 2.02 ± 1.23; *p* = 0.986).

### 3.3 Radiographical bone loss

In the OAOT group, the mean radiographical bone loss amounted to 1.55 ± 1.35 mm (min.: −0.10, max.: 5.99, median 1.39 mm) after a mean loading time of 9.23 ± 3.23 years (min.: 3.62, max.: 15.57, median 8.68 years).

For the AR group, the corresponding values of 2.93 ± 1.89 mm (min.: 0.25, max.: 7.43, median 3.06 mm) were noted after a mean loading time of 11.22 ± 2.39 years (min.: 6.89, max.: 14.38, median 11.23 years).

The linear regression revealed significant differences between OAOT and AR groups regarding radiographical bone loss (95% CI 0.25; 2.51, *p =* 0.0190). The estimated mean bone loss is in the AR group by 1.38 mm larger than in the OAOT group (Table 4).

**TABLE 4:** Bone loss.

	OAOT	AR	*p* value (estimate with 95% CI)
Bone loss (mm)			
Mean ± SD	1.55 ± 1.35	2.93 ± 1.89	0.019 (1.38 [0.25, 2.51])
Min	−0.10	0.25	
Max	5.99	7.43	
Median	1.39	3.06	

## 4 DISCUSSION

This retrospective study aimed at evaluating the effects of the repeated abutment replacement (i.e., OAOT vs. conventional AR protocol) on the prevalence of peri‐implant diseases over the mean follow‐up period throughout the follow‐up period of 10.2 ± 2.8 years.

Based on the present data analysis, 0.0% and 5.6% in the OAOT group and 22.2% and 55.6% of implants were diagnosed with peri‐implant mucositis and peri‐implantitis, respectively. As such, the abutment replacement was found to be a significant predictor for the increased prevalence of peri‐implant diseases, that is, peri‐implant mucositis and peri‐implantitis (*p <* 0.001). Furthermore, the probability of the diagnosis of peri‐implant mucositis and peri‐implantitis increased with the increased number of abutment replacements (OR: 6.13; 95% CI [2.61, 14.39]). To the authors' best knowledge, this is the first study evaluating the influence of the repeated replacement of the abutment upon the long‐term prevalence of peri‐implant diseases.

The prevalence of peri‐implant diseases obtained in the present analysis corroborates the results of previous studies that, however, did not specify the number of abutment replacement.^14^, ^15^ Furthermore, variations in the case definition applied for peri‐implant diseases do not allow for a direct comparison of the present data with those reported in the aforementioned studies. Nonetheless, one previous cross‐sectional analysis employing the same surgical protocol (i.e., subcrestal implant placement) and similar disease definitions, reported on the prevalence of peri‐implant mucositis and peri‐implantitis of 62.6% and 7.5% of the implants, which is comparable to the current outcomes. However, as noted above, the number of abutment replacements was not specified by the authors.

It should be noted that in the present retrospective study, the absolute PPDs were not included in the definition of peri‐implant diseases because an implant system with a wide horizontal offset was investigated, which may subsequently lead to a high risk of false negative measurements when the probe stops on top of the offset instead of following the implant body. This should be considered a limitation of the present study. In fact, based on the current recommendations, for the diagnosis of peri‐implant diseases, not the absolute PPD values, but rather the changes in the PPD values over time needs to be considered.^2^, ^16^ Furthermore, in this study, a threshold of 1.5 mm was considered for the assessment of bone loss to account for radiographic measurement errors.^12^

Even though the statistical analysis failed to reveal a significance for the factor keratinized mucosa (*p* = 0.986), a recent systematic review concluded that a width of less than 2 mm of keratinized mucosa was associated with a higher risk of peri‐implant disease.^17^ The study population, consisting of cases reconstructed with a telescopic retention mode, might have a favorable possibility for plaque control due to the round‐shaped surfaces of the primary crowns without undercuts. This could lead to similar results as presented by Lim et al., who concluded that the amount of keratinized mucosa may not influence peri‐implant health in compliant patients.^18^

Multiple abutment replacements were associated with a statistically significant increased peri‐implant bone loss when comparing to the placement of the final abutment immediately after the implant insertion (2.93 ± 1.89 vs. 1.55 ± 1.35 mm; *p =* 0.0190). These results are supported by the findings of a previous meta‐analysis assessing the effect of one‐time abutment placement on marginal bone levels at platform‐switching implants.^19^ In particular, the weighted mean difference in marginal bone loss amounted to 0.19 mm (95% CI, 0.06–0.32), thus confirming significantly greater bone loss in cases of multiple abutment disconnections/reconnections.^19^

Furthermore, the results from the present study are in agreement with those reported in previous studies employing the effect of one‐time abutment placement on marginal bone level at platform‐switching implants.^10^, ^20^ In particular, a significantly greater mean bone resorption was observed for those implants subjected to abutment replacement (control connection and disconnection of the healing abutment: 1.24 ± 0.79 mm, test [one‐time abutment placement]: 0.61 ± 0.40 mm) (*p =* 0.028) at 6 months post surgery.^10^ Additionally, there was a statistically significant difference in bone resorption changes around implants immediately loaded and restored using definitive abutments (DA) versus provisional abutments (PA) at the 6‐month (*p* < 0.001) and the 12‐month (*p* < 0.001) follow‐up: 0.294 mm (CI 95% 0.276; 0.312) and 0.341 mm (CI 95% 0.322; 0.36), respectively.^20^ As opposed to the aforementioned study, the present retrospective study reported on the amount of the bone loss after a mean time of 10.2 ± 2.8 years. The protective role of the use of platform‐switched implants was previously demonstrated in a preclinical animal study with a mean vertical bone resorption of 0.24 mm in the platform‐switched group versus 1.09 mm in the nonplatform‐switched implant group after 4 abutment dis‐ and reconnections (*p* < 0.05).^21^

On the other hand, one recent randomized clinical trial contradicted the aforementioned findings of the influence of abutment replacement upon marginal bone stability by stating that the “One Abutment‐One Time” concept does not reduce peri‐implant bone loss compared to the connection‐disconnection technique. In fact, the authors observed the height of the abutment being associated with bone stability, with increasing abutment height being related to marginal bone stability.^22^

In the present study, abutments in the control group were replaced up to six times (1 patient, 3 abutment changes in average) which is well in line with other preclinical or clinical studies where abutments were removed once,^10^ up to three,^22^ four^20^, ^21^, ^23^ or five times.^24^ Numerous abutment replacements might result in a weaker soft tissue seal due to an injury to the peri‐implant soft tissue. Already in 1997, the results of Abrahamsson et al. indicated that the dis‐ and subsequent reconnection of abutments compromised the mucosal barrier and resulted besides marginal bone resorption in a more apical extension of the junctional epithelium, resulting in a displacement of the sub‐epithelial connective tissue zone.^24^ These findings were supported by similar results of Becker et al. in 2014.^9^ In fact, a disruption of the mucosal seal, an apical displacement of the long junctional epithelium and subepithelial connective tissue zone were shown to be directly associated with abutment dis−/re‐connection. As a consequence, dimensional changes of peri‐implant soft and hard tissues were observed.^9^

In this context, minimizing the number of abutment dis‐ and reconnections would be beneficial to ensure minimal disruption of peri‐implant tissue and to maintain the marginal bone level.^25^

When interpreting the current findings in comparison to the papers cited in this discussion, it must be kept in mind that different implant placement protocols (i.e., immediate/early/delayed), various loading protocols, implant and abutment designs, prosthesis retentions (fixed/removable), and follow‐up times were used, which in fact might have influenced the obtained outcomes.

Other limitations of the current study are the use of panoramic radiographs for the inter‐proximal bone level analysis due to the retrospective nature of the investigation, the absence of a sample size calculation and, in this context, the different group sizes in the test and control groups, and the inherent nature of retrospective studies. Furthermore, one has to keep in mind a wide range of follow‐up periods (i.e., 3.62–15.57 years [OAOT group], respectively 6.89–14.38 years [AR group]), which in turn might have influenced the obtained outcomes. As shown by a former clinical analysis, vertical soft tissue thickness appears to be an important factor influencing the amount of bone loss during the initial bone remodeling.^26^ In the present analysis, however, the soft tissue thickness was not evaluated. Finally, it might be speculated that the depth of implant placement may further have an impact on the extent of marginal bone stability and peri‐implant tissue health. The present data set is based on subcrestally inserted implants. However, in terms of peri‐implant tissue health, a recent cross‐sectional analysis reported comparable prevalence of peri‐implant diseases at subcrestally positioned implants^14^ compared to equi‐ or supracrestal implant placement.^27^, ^28^

Considering the aforementioned limitations, randomized controlled trials focusing on the role of abutment changes in peri‐implant disease development could be helpful to reinforce clinical implications in implant prosthodontic concepts.

## 5 CONCLUSION

Within the limitations of this retrospective study, it was concluded that the one‐abutment one‐time concept was associated with lower prevalence for peri‐implant diseases. Thus, therapy concepts avoiding abutment changes seem to be beneficial.

## AUTHOR CONTRIBUTIONS

M.K. and P.W. conceived the ideas; L.G. collected the data and wrote the manuscript; ID analyzed the data and designed tables; M.K. and A.B. led the writing; P.H and A.B. gave support in the implementation and revision.

## CONFLICT OF INTEREST STATEMENT

The authors declare no conflict of interest.

## Data Availability

The data analyzed during the current study are available from the corresponding author on reasonable request.
